# Uncertainties in Measuring Populations Potentially Impacted by Sea Level Rise and Coastal Flooding

**DOI:** 10.1371/journal.pone.0048191

**Published:** 2012-10-24

**Authors:** Pinki Mondal, Andrew J. Tatem

**Affiliations:** 1 Department of Geography, University of Florida, Gainesville, Florida, United States of America; 2 Emerging Pathogens Institute, University of Florida, Gainesville, Florida, United States of America; 3 Fogarty International Center, National Institutes of Health, Bethesda, Maryland, United States of America; Kenya Medical Research Institute - Wellcome Trust Research Programme, Kenya

## Abstract

A better understanding of the impact of global climate change requires information on the locations and characteristics of populations affected. For instance, with global sea level predicted to rise and coastal flooding set to become more frequent and intense, high-resolution spatial population datasets are increasingly being used to estimate the size of vulnerable coastal populations. Many previous studies have undertaken this by quantifying the size of populations residing in low elevation coastal zones using one of two global spatial population datasets available – LandScan and the Global Rural Urban Mapping Project (GRUMP). This has been undertaken without consideration of the effects of this choice, which are a function of the quality of input datasets and differences in methods used to construct each spatial population dataset. Here we calculate estimated low elevation coastal zone resident population sizes from LandScan and GRUMP using previously adopted approaches, and quantify the absolute and relative differences achieved through switching datasets. Our findings suggest that the choice of one particular dataset over another can translate to a difference of more than 7.5 million vulnerable people for countries with extensive coastal populations, such as Indonesia and Japan. Our findings also show variations in estimates of proportions of national populations at risk range from <0.1% to 45% differences when switching between datasets, with large differences predominantly for countries where coarse and outdated input data were used in the construction of the spatial population datasets. The results highlight the need for the construction of spatial population datasets built on accurate, contemporary and detailed census data for use in climate change impact studies and the importance of acknowledging uncertainties inherent in existing spatial population datasets when estimating the demographic impacts of climate change.

## Introduction

The estimation of sizes of populations at risk (PAR) is increasingly being undertaken to guide strategic decision making and policy. PAR in terms of natural and manmade disasters [1], [2], hunger [3] and disease [4]–[8], for example, are now regularly estimated. Estimation of the likely impacts of climate change is becoming increasingly central to guiding strategic planning for mitigation of its effects and a key part of such impact evaluations is estimation of PAR.

These have included estimates of numbers impacted by flooding [9], [10], water shortages [11] and a variety of other hazards [12]. Approaches for deriving these estimates are increasingly making use of our improved abilities to produce detailed spatial datasets of climate change related phenomena and impacts, and overlaying these datasets on large area gridded population distribution datasets to calculate total numbers of people impacted.

**Figure 1:**
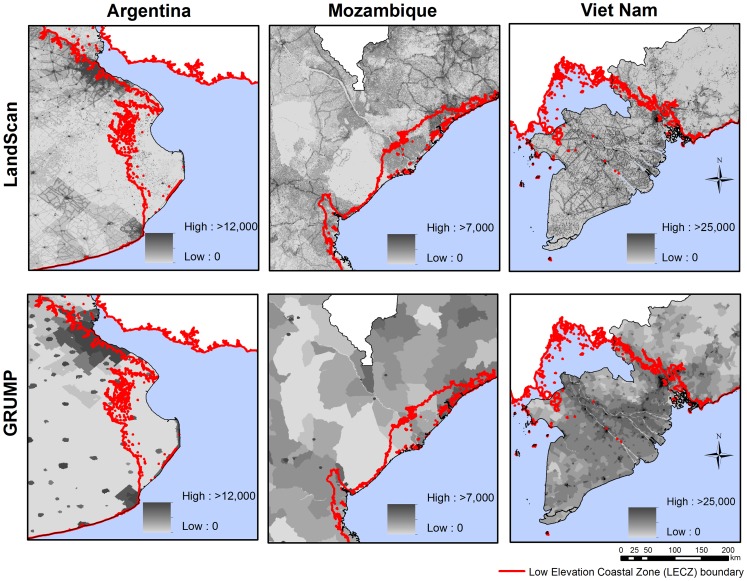
*Population distributions for east Argentina, east Mozambique and south west Viet Nam.* This figure illustrates population distributions in 3 countries as mapped by LandScan 2008 and GRUMP version 1. Values represent population counts per pixel. The low elevation coastal zone (LECZ) boundary is shown in red.

Approaches that are based on cartographic derivations of PAR are reliant on the accuracy of both the phenomena being mapped and the gridded population dataset. While the accuracies and uncertainties inherent in the development of climate change scenarios, and the mapping of their impacts are commonly debated and accounted for in impact studies, the accuracy of the accompanying population dataset used is rarely discussed, nor the impact on results of the choice of one dataset over another considered. Existing global population distribution datasets are built on databases of census data of varying year and resolution [13]. Moreover, spatially detailed contemporary census data is often not available for many low-income countries [13], [14], and therefore, global population datasets are often based on census data over 10 years old with counts reported for coarse administrative units [13]. These differing years of census data, when used along with a variety of intercensal growth rates, produce different input datasets to the global mapping projects that show large variations in population sizes and spatial distributions. These data are then disaggregated from population counts within administrative units to grids, using differing modeling rules. The most contemporary, detailed and widely used of these datasets are LandScan [15] and the Global Rural Urban Mapping Project (GRUMP) [16]. LandScan and GRUMP have been preferred for PAR estimates by many previous studies due to their finer spatial resolutions (30 arcseconds latitude/longitude grid or ∼1 km at the equator), and more regular updates and incorporation of detailed ancillary data for modeling than the other available gridded population datasets. LandScan disaggregates census data based on various weightings derived from land cover data, proximity to roads, slope, and populated areas/points [15]. While based on residential census population counts, the grid cells of LandScan data represent ‘ambient’ population distribution integrating diurnal movements and collective travel habits into a single measure (see Materials and Methods for details), and national totals are adjusted to match those reported by the US Census Bureau. GRUMP uses night-time light satellite data as a proxy for urban areas, reallocating census count data within administrative boundaries based on rural-urban extents [16], and adjusting national totals to those made by the United Nations Population Division (UNPD). The cumulative effect of differences in input data, modeling approaches and adjustments to totals leads to some large differences in estimated population distributions (as illustrated by Fig 1), which in turn have effects on applications, as shown for estimates of PAR of disease [8], [14]. Both LandScan and GRUMP have been widely used to estimate the size of PAR of sea level rises and coastal flooding, without consideration of these differences.

**Figure 2:**
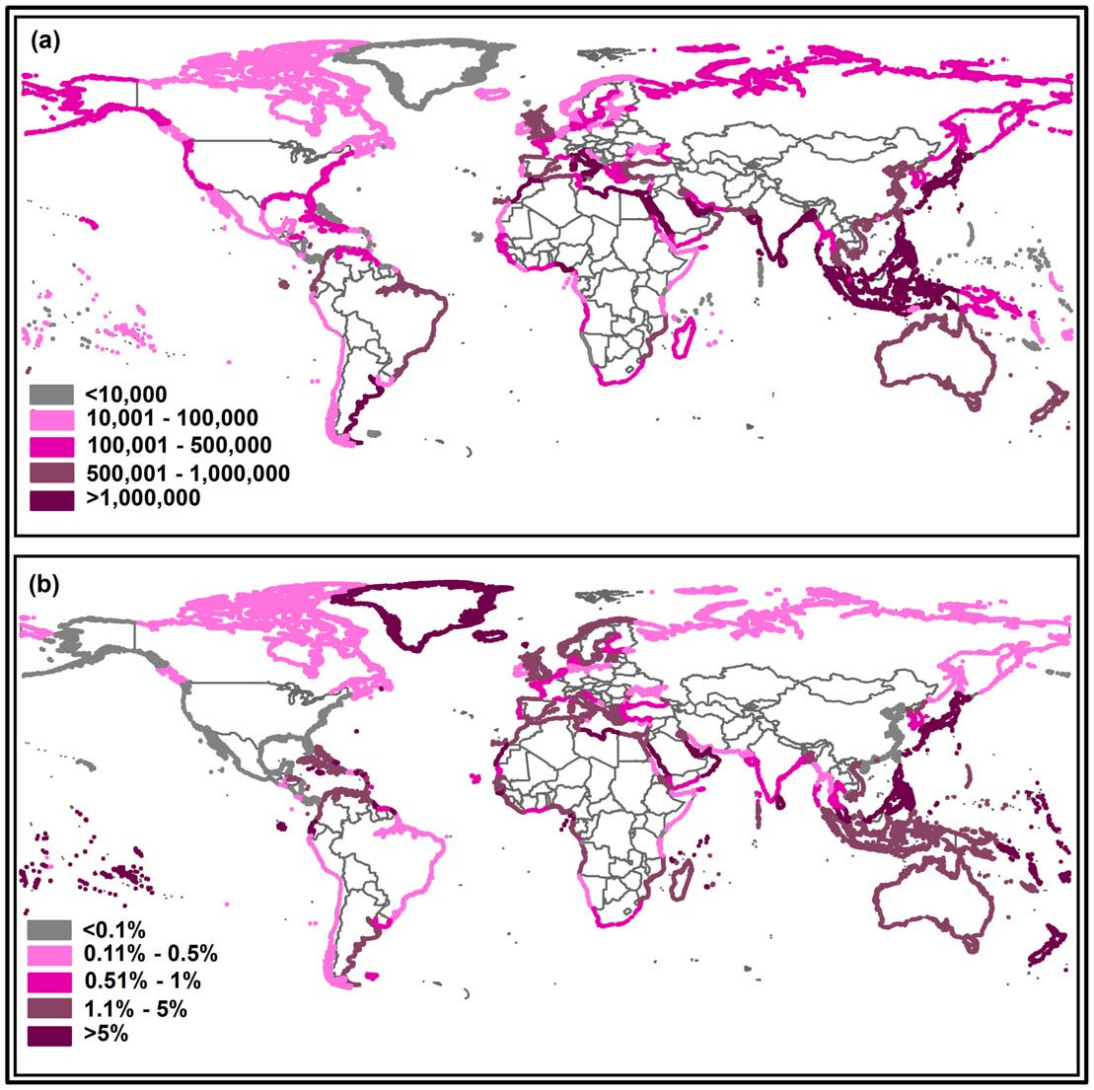
*Variability in population at risk (PAR) estimates.* This figure highlights the differences in PAR estimates residing in low elevation coastal zones (LECZ) across the world achievable through switching between LandScan and GRUMP: (a) absolute differences in 2008 between LandScan and GRUMP PAR estimates, and (b) percentage change in PAR from 2008 national population totals defined by the United Nations Population Division.

Global sea level has risen through the 20^th^ century and is expected to rise up to ∼60 cm by 2100 [17]. Sea level rise (SLR) can also be triggered by extreme climate changes, such as the potential collapse of the West Antarctic Ice Sheet, and may even reach up to 6 m by 2130 [18].

While such high impact climate-induced events have very low occurrence probability and SLR above 2 m by 2100 likely cannot be justified [19], extreme SLR predictions are often integrated in studies estimating global impacts of climate change to inform policy-makers [20], [21]. Coastal flooding and storms during events are expected to occur with greater frequency and intensity through climate change. Moreover, geographic variation in SLR resulting from non-uniform distribution of temperature, salinity, and associated surface ocean circulation changes are likely to affect low elevation coastal zones (LECZ) [22]. Predicted SLR and increasing occurrences of coastal flooding have prompted a set of studies to quantify impact on vulnerable LECZ population through PAR estimates [20], [21], [23]–[25]. These estimates are generated by the cartographic overlay of gridded population distribution datasets and LECZ footprints (defined as low-lying land areas contiguous to the coastal boundary), where coastal areas below 10m of elevation are considered a conservative estimate of the vulnerable zones [18], [21]. Figure 1 highlights the differences in estimated LECZ population distributions for three example regions that result from the differing modeling procedures used by GRUMP and LandScan. GRUMP has been used to determine the population and urban settlement patterns in LECZ [21], and to map climate change risks to populations in Africa, Asia and South America [23]. Similarly, LandScan has been utilized to estimate PAR due to climate change along the US coast [24], to estimate land area loss and population affected due to inundation scenarios [25], and to assess SLR effects on populations living on deltas across a range of geographic, and economic conditions [20]. Finally, influential Intergovernmental Panel on Climate Change (IPCC) reports on climate change impacts [26] refer to evidence from sets of studies that base PAR estimates on either GRUMP or LandScan with no acknowledgment of the possible uncertainties that arise. All of these studies rely heavily on the accuracy of (i) the coastal mapping, and (ii) the population distribution datasets.

**Table 1:** Continent-wise low elevation coastal zone (LECZ) population estimates derived from LandScan 2008 and GRUMP (projected to 2008) datasets.

Continent	LECZ population estimates: LandScan	LECZ population estimates: GRUMP	Total 2008 population estimates by UNPD of LECZ countries	LECZ population estimates as % total UNPD population: LandScan	LECZ population estimates as % total UNPD population: GRUMP	% difference in LECZ population derived from LandScan and GRUMP datasets
Africa	63,050,042	57,096,275	752,731,674	8.38	7.59	0.79
Americas	60,548,793	58,705,036	904,609,518	6.69	6.49	0.20
Asia	550,417,035	531,441,504	3,935,404,359	13.99	13.50	0.48
Europe	47,828,449	45,937,995	665,829,990	7.18	6.90	0.28
Oceania	4,168,768	2,546,088	34,605,327	12.05	7.36	4.69

Relative differences in LECZ population estimates are also reported as percentage of the total population of the LECZ countries from each of these continents as estimated by the United Nations Population Division (UNPD). A detailed list of all countries has been provided in Table S1.

A recent study showed that elevation data from different sources introduces variations in PAR estimates [27]. Our objective in this study is to demonstrate the variability in PAR estimates that can be obtained through using different population datasets, rather than presenting PAR estimates in a plausible sea level rise scenario. In order to achieve this goal, we maintain the elevation data as constant, and examine the effects of varying the population distribution data. We quantify the differences in PAR estimates derived from LandScan and GRUMP to illustrate the uncertainty introduced by the choice of dataset. We use global population distribution datasets for LandScan and GRUMP in combination with a satellite-derived dataset outlining the boundaries of land area contiguous with the coastline up to 10m of elevation to extract population total estimates within the LECZ (see Materials and Methods for details). The estimated totals using LandScan and GRUMP are compared at continental and national levels to assess the size of PAR variations achievable through switching population dataset, and we calculate both absolute differences between LandScan and GRUMP and relative differences between the two datasets.

**Table 2:** Country-level differences between population at risk (PAR) estimates achievable through switching between LandScan and GRUMP.

Country	Difference in PAR estimates as % of national population estimates (UNPD)	National population estimates for 2008 (UNPD)
***Top 10 countries with the largest PAR disparities***		
Saint Pierre et Miquelon (Americas)	47.12	6,036
Wallis and Futuna (Oceania)	44.65	15,297
Samoa (Oceania)	43.20	177,883
Guyana (Americas)	40.88	757,659
United Arab Emirates (Asia)	39.77	3,683,453
Anguilla (Americas)	35.66	14,277
British Virgin Islands (Americas)	31.07	22,495
French Polynesia (Oceania)	29.43	265,497
Tuvalu (Oceania)	28.74	9,946
Tonga (Oceania)	28.54	102,737
***Top 10 countries based on combined ranking of large PAR disparities and large population (>1,000,000)***		
United Arab Emirates (Asia)	39.77	3,683,453
Gambia (Africa)	20.12	1,656,103
Libya (Africa)	19.45	6,297,761
Oman (Asia)	18.48	2,751,575
Qatar (Asia)	17.03	1,111,849
New Zealand (Oceania)	13.79	4,209,284
Guinea-Bissau (Africa)	13.69	1,580,870
Singapore (Asia)	10.87	4,508,366
Sri Lanka (Asia)	7.65	20,005,855
Philippines (Asia)	6.59	90,438,674

The PAR differences are reported here as proportions of the total national population of the corresponding countries as estimated by the United Nations Population Division (UNPD) for 2008. The top 10 countries with the highest PAR disparity are listed, alongside the top 10 by PAR disparity for countries with populations over one million. A detailed list of all countries has been provided in Table S1.

## Materials and Methods

### Population datasets

The most recent (at the time of writing) population count datasets from LandScan (2008 version) [28] and the Global Rural Urban Mapping Project (GRUMP) (2000 version 1) [29] were obtained. Both of these datasets have a spatial resolution of 30 arcseconds (∼1 km at the equator). Both LandScan and GRUMP are based upon census population counts – the main difference between the two is the year and administrative levels of input census data used, and the modeling procedures used to disaggregate these data. LandScan disaggregates annual midyear sub-national population estimate data based on weightings derived from land cover, roads, slope, urban areas, village locations, and high resolution imagery analysis; hence the population distribution surface is a highly modeled one that represents ‘ambient’ population distribution. The GRUMP suite of data products were developed in an effort to reallocate census population counts to urban and rural areas, and not just areal weighting of census counts to a grid as followed in the production of the Gridded Population of the World (GPW) dataset [30]. Unlike GPW, GRUMP is a ‘lightly modeled’ dataset that not only uses areal weighting to redistribute population counts from administrative polygons (census counts from census boundaries) to a uniform quadrilateral grid, but also reallocates urban population based on night-time lights. Since GRUMP represented population in 2000 and LandScan represented 2008, two methods for producing temporally comparable datasets were used: (i) The LandScan and GRUMP datasets were projected to common years (GRUMP to 2008 to match LandScan, and also LandScan to 2000 to match GRUMP) to ensure comparability by applying national, medium variant, intercensal growth rates by country [31], following methods described previously [32] and also undertaken in other LECZ studies [18], [20] (ii) We calculated national level population totals for LandScan 2008 and GRUMP 2000, using their respective national boundary definitions, and adjusted national totals in GRUMP 2000 to match those of LandScan 2008, and vice-versa. The differences in country-level percentage differences between LECZ estimates for the two time periods were found to be statistically insignificant, illustrating that the differences in LECZ PAR found between datasets are largely insensitive to different projection methods used in this study. Each of these adjustment approaches, however, ultimately contributes to additional uncertainty in the PAR estimates, a fact that is also rarely acknowledged in previous studies. While not ideal, the use of national level growth rates to project or backcast datasets to specific years is regularly undertaken, since the availability of LandScan and GRUMP for specific years often does not meet the needs of the researchers using these data. Examples of studies that have undertaken similar approaches can be found on the GPW/GRUMP website [29]. By undertaking these different methods of projection and producing comparable datasets, and assessing the differences in PAR estimates produced, the sensitivity and contribution to any PAR size differences of the different comparison approaches could be assessed.

### Elevation Datasets

The global LECZ footprint used in this study is derived from the SRTM30 Enhanced Global Map, which is based on raw SRTM data, but is enhanced with the U.S. Geological Survey's GTOPO30 and ocean bathymetry data from ETOPO2 [18]. This enhanced global dataset, developed by ISciences, LLC, Ann Arbor (2003), has a vertical resolution of 1 m and spatial resolution of 30 arcseconds (∼1 km at the equator), and corrects for the data gap and inaccuracies of raw SRTM data. The LECZ layer includes land areas, 10 m and below, contiguous to coastal boundaries, and expands down to −4000 m to include areas below sea level. The expansion of the LECZ footprint beyond the coastal boundary ensures inclusion of populations residing below sea level and protected by levees, and also addresses issues regarding mismatches in coastal boundaries between population datasets, especially in small island countries. In most countries, LECZ is much less than 100 km in width, except for the mouths of major rivers such as the Amazon in Brazil.

### PAR extraction

The LECZ footprint was used to extract LECZ population data from LandScan, and GRUMP. This extraction method involved cartographic overlaying of the LECZ footprint and population grids in a Geographic Information System (GIS) resulting in raster layers for LECZ population, one each for LandScan and GRUMP. The files accompanying GRUMP and LandScan that define country cell allocations across the world were used, respectively, to summarize population data for LECZ countries, i.e. those not land-locked. Absolute differences between LandScan and GRUMP population estimates (using datasets adjusted to be comparable using the different approaches outlined above) were then calculated for the LECZ countries, and later used to calculate the differences, both at the country and continent levels, as the percentage of the total UNPD 2008 populations. The percentage differences calculated using the GRUMP and LandScan datasets adjusted in differing ways (projections to 2000 and 2008, national population total matching) showed no overall significant differences (Mann-Whitney U-test, p<0.01 in all cases), suggesting that projection method contributes statistically insignificant differences relative to the differences introduced through differing input datasets and modeling approaches used between GRUMP and LandScan. This is consistent with previous findings [13].

## Results and Discussion

At the continental level there is little variation in PAR estimates derived from LandScan and GRUMP (Table 1), however such summarizations mask some substantial variations at the country level. Several countries from all the five continents exhibit PAR differences between LandScan and GRUMP of over 5% of the UNPD national population estimates (Fig. 2). Most of these countries are, unsurprisingly, small islands, with their entire land area in the LECZ. Eight out of the top ten countries with the largest differences in estimates are small island countries (Table 2), with five of them having total population of less than 25,000. These countries exhibit over 25% difference in their PAR estimates due to choice of population dataset , with the largest difference being approximately 47% for the American island of Saint Pierre et Miquelon. While the spatial datasets used in this study have similar coastal boundaries for the vast majority of regions, small islands often display inconsistencies due to a mismatch in cell gridding used in the two population datasets. Since the LECZ used in this study expands beyond the coastal boundaries to include areas down to −4000 m, it addresses such mapping inconsistencies in small island countries. Inconsistencies in definitions of global administrative boundaries, however, inevitably introduce uncertainties into PAR estimates – a fact rarely acknowledged.

Census datasets used to construct both GRUMP and LandScan for Europe are of similar, detailed resolution, meaning that the difference in modeling approach taken by GRUMP and LandScan generally have little impact on output population distributions, and thus, country-level discrepancies between PAR estimates are small (Fig. 2). Similarly, high-resolution census tract-level count data used as input resulted in very similar population distributions for the US, as quantified by LandScan and GRUMP, producing only 0.1% differences between the two PAR estimates, despite substantial LECZ populations in excess of 25 million today. The PAR estimates for African countries, where input census data varies considerably in resolution and quality [13], exhibit much larger differences however (Fig. 2). Moreover, many of the Central and South American countries such as Argentina, Belize, Colombia, Cuba, Haiti, Honduras, and Venezuela have a 1%-3% relative difference in PAR achievable through switching between LandScan and GRUMP. While these proportions might seem low, for a country such as Argentina with relatively large coastal populations, this relative difference translates to over a million people. When those countries with a population greater than 1 million were ranked from largest to smallest by differences in PAR estimates, nine out of ten of the top ten countries were either Asian or African, showing differences ranging from 6% to 39% of their total population (Table 2). Absolute differences of more than a million people for 2008 were found for many countries, including Indonesia and Japan, with each of these showing a difference of greater than seven million through estimating populations living in LECZ using GRUMP and LandScan. Moreover, for India, a country with high coastal population densities, a percentage difference of just 0.5% translates to over six million people.

Ideally, identifying which existing population dataset produced the most accurate distributions within LECZ would provide clear guidance for future selection of datasets for PAR estimation. Determining this remains a difficult task, however, because if detailed population distribution data exists, it is often used as input data to the global population datasets themselves. Previous studies have, however, attempted to assess mapping accuracies between population datasets for individual countries, and often found large variations and inconsistent results between countries in terms of which dataset proved to be the most accurate, largely determined by the resolution and age of input census data [32]–[34]. Most countries collect census data once a decade, and such data are often not made available matched to reliable spatial boundary data. Moreover, many low incomes countries – particularly in sub-Saharan Africa – have not conducted full censuses for over 15 years. At finer spatial scales, uncertainties arise due to the daily dynamics of populations within urban environments – e.g. data on residential populations excludes large commercial and industrial areas of the urban environment which are more populated during daylight hours, thus underestimating high population densities in coastal urban areas for much of the day. From this perspective, it may not be prudent to use residential census data as a reference to produce error statistics for ambient population data, especially in coastal PAR studies where the temporal component of PAR is of importance. Of perhaps greater importance is the lack of information in existing spatial population datasets on the attributes of populations mapped, such as age and sex. Different population groups are more vulnerable to the effects of climate change, disasters and disease than others, yet existing datasets only provide information on total population counts [8].

With predicted SLR in the coming decades posing multiple threats to vulnerable coastal populations, PAR assessments are being increasingly undertaken [35]. Spatial population datasets have played an important role in previous LECZ PAR estimate studies, as well as in numerous other fields of research [14], [16], [36]. Our findings here highlight that even today with increasingly accurate, detailed, and reliable spatial data on climatic and environmental variables, our knowledge of human population distributions – especially in low income regions of the world – can be surprisingly limited [13]. With the advancement of theory and computational capabilities, future work on spatial population datasets should ideally focus on integrating robust handling of uncertainties into demographic database construction methods as a priority [8]. Moreover, dataset producers could also consider the modification and provision of open-access availability of modeling techniques so that improved input data can be more easily incorporated into datasets, even when made available after the modeled products have been generated. Models for population allocation need to be critically evaluated and revised on a regular basis, and perhaps most importantly, the most contemporary and spatially detailed population data should ideally be shared at all times, with full metadata documenting production and any accuracy information. While efforts to improve spatial population data have been started through differing projects [37], [38], these remain small in scope and capacity. Hence, in the absence of global population databases with greater precision, studies utilizing a particular dataset should acknowledge how the inherent uncertainties of the input data and method will likely affect conclusions.

## Supporting Information

Table S1 **Country-wise low elevation coastal zone (LECZ) population estimates derived from LandScan 2008 and GRUMP (projected to 2008) datasets.** Relative differences in LECZ population estimates are also reported as percentage of the total population of the LECZ countries from each of these countries as estimated by the United Nations Population Division (UNPD).
(XLSX)
